# Zespół łamliwego Chromosomu X i Choroby *FMR1*-zależne - Postępowanie Diagnostyczne Na Podstawie Doświadczeń własnych

**DOI:** 10.34763/devperiodmed.20182201.2232

**Published:** 2018-04-12

**Authors:** Aleksandra Landowska, Sylwia Rzońca, Jerzy Bal, Monika Gos

**Affiliations:** 1 Zakład Genetyki Medycznej, Instytut Matki i Dziecka, Warszawa, Polska

**Keywords:** *FMR1*, PCR, TP-PCR, MS-MLPA, mutacja dynamiczna, *FMR1*, PCR, TP-PCR, MS-MLPA, dynamic mutation

## Abstract

Obecność mutacji dynamicznej w genie FMR1 zlokalizowanym na chromosomie X (Xq28) stanowi główną przyczynę wystąpienia zespołu łamliwego chromosomu X. Ze względu na fakt, że jest to jedna z częściej identyfikowanych chorób, w których przebiegu stwierdza się niepełnosprawność intelektualną i zaburzenia ze spektrum autyzmu, badanie w kierunku tej choroby jest elementem rutynowej diagnostyki genetycznej u pacjentów z tego typu zaburzeniami. W chwili obecnej standardem diagnostycznym są badania molekularne oparte o technikę PCR umożliwiające identyfikację alleli z zakresu prawidłowego (do 54 powtórzeń CGG, w tym alleli z zakresu tzw. „szarej strefy” – 45-54 powtórzenia CGG), premutacji (55-200 powtórzeń CGG) i pełnej mutacji (>200 powtórzeń CGG).

W artykule przedstawiono podstawowe metody stosowane w diagnostyce molekularnej zespołu łamliwego chromosomu X i innych chorób FMR1-zależnych, tj. test przesiewowy z analizą GeneScan, test TP-PCR oraz metody wykorzystywane do analizy metylacji. Omówiono zalety i ograniczenia poszczególnych technik oraz sposób interpretacji uzyskiwanych wyników. Przedstawiono także schemat postępowania diagnostycznego stosowany w Zakładzie Genetyki Medycznej IMiD, zgodny z zaleceniami European Molecular Genetics Quality Network.

## Abstract (en)

The presence of dynamic mutation in the FMR1 gene localized on the X chromosome (Xq28) is the major cause of Fragile X syndrome. As this syndrome is quite frequently diagnosed in patients with intellectual disability and autism spectrum disorders, the genetic testing of the FMR1 gene is a routine procedure performed in these patients. Molecular methods based on the PCR technique are used commonly, as they allow to identify normal (up to 54 CGG repeats, including grey zone alleles – 45-54 CGG repeats), premutation (55-200 CGG repeats) and full mutation (>200 CGG repeats) alleles.

The article presents the basic methods used in the molecular diagnosis of Fragile X syndrome and other FMR1-related disorders. The following methods are presented: a screening test with GeneScan analysis, TP-PCR based tests and methods used for methylation analysis. Their pros and cons, as well as the resulting interpretation are discussed. Moreover, there is a presentation of the molecular diagnostic scheme following European Molecular Genetics Quality Network guidelines used in the Department of Medical Genetics.

Zespół łamliwego chromosomu X (FXS) jest określany mianem modelowej choroby genetycznie uwarunkowanej. Jest to choroba sprzężona z płcią − gen *FMR1*, którego mutacje identyfikowane są u pacjentów z FXS, zlokalizowany jest na chromosomie X (Xq28). Ponadto u większości (>99%) chorych stwierdza się mutację dynamiczną – zwielokrotnienie powtórzeń trzynukleotydowej sekwencji CGG do zakresu powyżej 200 powtórzeń. Duże nagromadzenie powtórzeń CGG jest sygnałem do metylacji regionu 5’UTR genu *FMR1*, co blokuje ekspresję genu, skutkując brakiem białka FMRP w komórkach rozwijającego się organizmu [1, 2]. Jedna z hipotez sugeruje, że sygnałem do zablokowania ekspresji genu jest obecność krótkich niekodujących fragmentów RNA, powstałych w wyniku degradacji transkryptu *FMR1* zawierającego liczne powtórzenia CGG i tworzącego specyficzne struktury przestrzenne, określane mianem „szpilki do włosów”. Fragmenty RNA rozpoznawane są przez zależny od RNA inicjator represji genów, który przemieszcza się w region zawierający gen *FMR1* i inicjuje procesy hamujące jego ekspresję poprzez aktywację specyficznych białek (np. metylotranferazy DNA) [3].

Mutacja dynamiczna w genie *FMR1* powstaje w wyniku znacznego zwielokrotnienia powtórzeń CGG (tzw. ekspansja) w trakcie procesu replikacji DNA w komórkach rozrodczych. Ryzyko takiej ekspansji wzrasta wraz ze wzrostem liczby powtórzeń CGG u rodzica i jest znacząco wyższe dla alleli z zakresu premutacji (liczba powtórzeń CGG: 55-200). W przypadku alleli zawierających mniej niż 55 powtórzeń sekwencji CGG nigdy nie odnotowano ich zwiększenia do zakresu pełnej mutacji w jednym pokoleniu, nawet w przypadku alleli z zakresu tzw. „szarej strefy” (45-54 powtórzenia CGG). Dla stabilności liczby powtórzeń ma również znaczenie obecność w obrębie powtórzeń CGG stabilizujących sekwencji AGG. Wykazano, że im większa liczba powtórzeń AGG tym bardziej stabilne jest przekazywanie pomiędzy pokoleniami alleli o określonej długości. W allelach z zakresu premutacji/pełnej mutacji rzadko kiedy stwierdza się obecność sekwencji AGG [4, 5, 6, 7, 8].

Zespół łamliwego chromosomu X jest jedną z głównych genetycznych przyczyn wystąpienia niepełnosprawności intelektualnej (NI) i autyzmu, zaś badanie molekularne w kierunku identyfikacji mutacji dynamicznej w genie *FMR1* jest zlecane jako jedno z pierwszych badań diagnostycznych w przypadkach stwierdzenia NI, opóźnienia rozwoju psychoruchowego czy zaburzeń zachowania. Potwierdzenie rozpoznania klinicznego FXS jest podstawą do wdrożenia odpowiedniego postępowania terapeutycznego, które w przyszłości wraz z rozwojem nowych sposobów leczenia może mieć charakter spersonalizowany. Identyfikacja mutacji dynamicznej u probanta jest podstawą do objęcia pacjenta i jego rodziny opieką przez poradnię genetyczną. Osoby z premutacją w genie *FMR1* – nosiciele zespołu łamliwego chromosomu X, są potencjalnymi pacjentami poradni ginekologicznej i poradni neurologicznej, ze względu na ryzyko wystąpienia u nich zespołu przedwczesnego wygasania czynności jajników oraz zespołu drżenia i ataksji związanego z zespołem FXS (odpowiednio, FXPOI *– Fragile X-associated primary ovarian insufficiency* i FXTAS – *Fragile X-associated tremor/ ataxia syndrome*).[9] Stąd też istotne jest wdrożenie jak najbardziej optymalnych procedur diagnostycznych, które w sposób wiarygodny potwierdzą bądź wykluczą rozpoznanie zespołu łamliwego chromosomu X i innych chorób *FMR1*-zależnych.

Zakład Genetyki Medycznej Instytutu Matki i Dziecka jako pierwszy wprowadził metody umożliwiające identyfikację mutacji dynamicznej w genie *FMR1* w rodzinach z zespołem łamliwego chromosomu X. W latach 80-tych XX w. diagnostyka tej choroby opierała się na badaniach cytogenetycznych oraz analizie RFLP. Opisanie w 1991 r. mutacji dynamicznej w genie *FMR1* było początkiem rozwoju technik umożliwiających identyfikację alleli o określonej wielkości, począwszy od opracowania testu z wykorzystaniem hybrydyzacji genomowej typu Southern, przez metody oparte o technikę PCR, TP-PCR, skończywszy na dokładnym określeniu liczby powtórzeń z wykorzystaniem sekwencjonowania następnej generacji (NGS, PacBio; Tabela I). Wszystkie te metody (z wyjątkiem NGS) były stopniowo wdrażane w Zakładzie Genetyki Medycznej IMiD do rutynowej diagnostyki zespołu łamliwego chromosomu X, uwzględniającej również diagnostykę prenatalną choroby [10, 11, 12, 13, 14]. Artykuł ma na celu przybliżenie technik molekularnych stosowanych w diagnostyce zespołu łamliwego chromosomu X oraz chorób *FMR1*-zależnych oraz przedstawienie aktualnego schematu postepowania diagnostycznego.

## 1 Materiał do Badań Diagnostycznych w Kierunku Zespołu Łamliwego Chromosomu X

Podstawowym i jednocześnie najlepszym materiałem do wykonania postnatalnej analizy molekularnej w kierunku zespołu łamliwego chromosomu X oraz chorób związanych z obecnością premutacji w genie *FMR1* jest krew obwodowa pobrana na antykoagulant, najczęściej EDTA, z której izoluje się genomowy DNA. Pobrany materiał może być przechowywany przez krótki czas w temperaturze +4°C i w przeciągu 48 godzin powinien trafić do laboratorium diagnostycznego. Zgodnie z rozporządzeniem Ministra Zdrowia z dn. 19/08/2015 (poz. 1372)1Rozporządzenie Ministra Zdrowia z dnia 19 sierpnia 2015 r. zmieniające rozporządzenie w sprawie standardów jakości dla medycznych laboratoriów diagnostycznych i mikrobiologicznych (Dz. U. 2006, Nr 61, poz. 435 oraz z 2009 r. Nr 22, poz. 128); Załącznik 1. Standardy jakości dla laboratorium w zakresie czynności laboratoryjnej genetyki medycznej oraz laboratoryjnej interpretacji i autoryzacji wyniku badań. materiał do badań powinien zostać przesłany do laboratorium diagnostycznego wraz z odpowiednią kartą skierowania na badanie molekularne i podpisaną przez pacjenta lub jego opiekuna prawnego, deklaracją świadomej zgody na wykonanie badań molekularnych.

Krew obwodowa od pacjenta skierowanego na badanie genetyczne, w tym na badanie molekularne w kierunku FXS i chorób *FMR1*-zależnych, może być pobrana o każdej porze dnia, bez wcześniejszego przygotowania pacjenta. Ważne jest, aby w przypadku osób po transfuzji preparatu pełnej krwi, materiał do badań nie był pobrany wcześniej niż 2 miesiące po wykonanym zabiegu. Wynika to z tego, że w pobranym materiale mogą znaleźć się komórki dawcy, co zafałszuje wynik analizy molekularnej. Podobnie jest w przypadku osób, które przeszły allogeniczny przeszczep szpiku. U osób tych, źródłem materiału do badań molekularnych mogą być fibroblasty skóry czy wymaz materiału z jamy ustnej [2].

**Table I:** The summary of molecular methods used in Fragile X syndrome molecular diagnosis. Tabela I. Podsumowanie metod molekularnych stosowanych w diagnostyce zespołu łamliwego chromosomu X.

Metoda *Technique*	PCR *PCR*	PCR+GeneScan *PCR+GeneScan*	Hybrydyzacja *Hybridization*	TP-PCR *TP-PCR*	MS-MLPA *MS-MLPA*	MS-TP-PCR *MS-TP-PCR*	PCR-HRM *PCR-HRM*	NGS (PacBio) *NGS (PacBio)*
Ilość DNA *DNA* *amount*	min. 50ng	min. 50ng	10 ug	20 - 80ng	50-200ng	160 ng	100ng	>1.2pg
Metoda przesiewowa *Pre-screening* *test*	+	+	-	-	-	-	+	-
Dokładne określenie liczby powtórzeń CGG *Assessment* *of CGG* *repeat* *number*	-	+ (<130)	-	+ (<200)	-	+ (<200)	-	+
** Analiza metylacji *Methylation analysis*	-	-	+	-	+	+	-	?
Oznaczenie liczby przerw AGG *The number* *of AGG* *breaks*	-	-	-	+	-	-	-	+
Analiza mozaikowości somatycznej *Analysis* *of somatic* *mosaicism*	-	-	+	+	-	+	-	+
Analiza mozaikowości metylacyjnej ***Analysis*** ***of methylation*** ***mosaicism***	-	-	+	-	+	+	-	?

Procedura izolacji DNA zależy od laboratorium i może odbywać się w sposób konwencjonalny z wykorzystaniem metody wysalania DNA czy ekstrakcji w układzie fenol/ chloroform lub z wykorzystaniem metod opracowanych przez firmy biotechnologiczne. Metody te znacznie skracają czas potrzebny na izolację DNA i mogą być oparte o system kolumn zawierających odpowiednie złoże (np. kolumny z membranami krzemionkowymi lub złożami jonowymiennymi) czy system automatycznej ekstrakcji oparty na cząstkach magnetycznych.

W przypadku badań prenatalnych jako źródło DNA wykorzystuje się komórki płodu znajdujące się w płynie owodniowym, pobranym pomiędzy 14 a 20 tygodniem ciąży lub komórki trofoblastu, którego biopsję wykonuje się między 11 a 14 tygodniem ciąży [5].

## 2 Techniki Molekularne Stosowane Przy Identyfikacji Mutacji Dynamicznej w Genie *FMR1*

Identyfikacja mutacji dynamicznej w genie *FMR1* jako przyczyny wystąpienia zespołu łamliwego chromosomu X jest kluczowa dla diagnostyki tej choroby. Początkowo podstawową metodą była analiza kariotypu pacjenta, w trakcie, której poszukiwano charakterystycznego przewężenia w rejonie Xq27.3 na długim ramieniu chromosomu X. [15] Obecnie, analiza kariotypu została zastąpiona bardziej czułymi i specyficznymi badaniami molekularnymi, które zalecane są przez EMQN w diagnostyce molekularnej FXS, FXTAS i FXPOI. Większość stosowanych metod umożliwia jedynie identyfikację mutacji dynamicznej w genie *FMR1*. Identyfikacja rearanżacji chromosomowych np. rozległych delecji obejmujących gen *FMR1* czy mutacji punktowych wymaga zastosowania dodatkowych metod m.in. porównawczej hybrydyzacji genomowej do mikromacierzy czy sekwencjonowania genu metodą Sangera. Dlatego też metody te wykorzystywane sąw dalszych etapach procedury diagnostycznej [16].

### 2.1 Test przesiewowy PCR z analizą GeneScan

Metodą, która znacznie ułatwiła diagnostykę zespołu łamliwego chromosomu jest technika łańcuchowej reakcji polimerazy (PCR, ang. *polymerase chain reaction*), która niewątpliwie zrewolucjonizowała całą genetykę. Metoda opracowana w latach 80-tych przez K. Mullisa i wsp. umożliwia wykładnicze powielenie wybranego fragmentu genu, dzięki cyklicznej reakcji syntezy nici DNA. W trakcie jednego cyklu reakcji PCR określony fragment DNA jest kopiowany i staje się w kolejnym cyklu matrycą do syntezy nowej nici DNA. Jak łatwo obliczyć w reakcji składającej się z 35 cykli pojedynczy fragment DNA daje 2^35^ kopii. Każdy cykl w reakcji PCR składa się z trzech etapów: denaturacji (rozdzielenie podwójnej nici DNA na dwie pojedyncze), przyłączania specyficznych starterów do nici DNA oraz syntezy nowej nici DNA. Każdy z tych etapów odbywa się w określonej temperaturze (denaturacja – 95°C, przyłączanie starterów – temperatura specyficzna dla reakcji, zazwyczaj w zakresie 50-60°C i synteza – zazwyczaj 72°C), w związku, z czym wymaga użycia termocyklerów – urządzeń, które w sposób cykliczny generują zmiany w zakresie temperatur.

Produkt reakcji PCR staje się przedmiotem dalszych analiz, zarówno jakościowych (np. analiza sekwencji powielonego fragmentu), jak i ilościowych (ocena ilości produktu PCR np. ilościowy PCR w czasie rzeczywistym).

Amplifikacja fragmentu genu *FMR1* zawierającego powtórzenia CGG jest podstawowym badaniem w diagnostyce zespołu łamliwego chromosomu X, określanym często jako badanie przesiewowe (Rycina 1a). Należy wspomnieć, że powielenie ciągu jednakowych powtórzeń jest stosunkowo trudne – polimeraza stosowana w reakcjach PCR może „ślizgać się” po matrycy, w związku, z czym nie każdy powstały fragment jest dokładną kopią sekwencji DNA. Istnieje również pewien pułap liczby powtórzeń, który ogranicza możliwość powstania produktu –w przypadku sekwencji (CGG)^n^ jest to około 130 powtórzeń. Dodatkową trudnością w tej analizie jest fakt, że powielany fragment jest bogaty w pary GC, co zmniejsza wydajność reakcji PCR. Stosuje się, zatem odpowiednio długi czas denaturacji, jak również związki ułatwiające działanie polimerazy (np. betaina lub DMSO) [2, 5].

**Ryc. 1:**
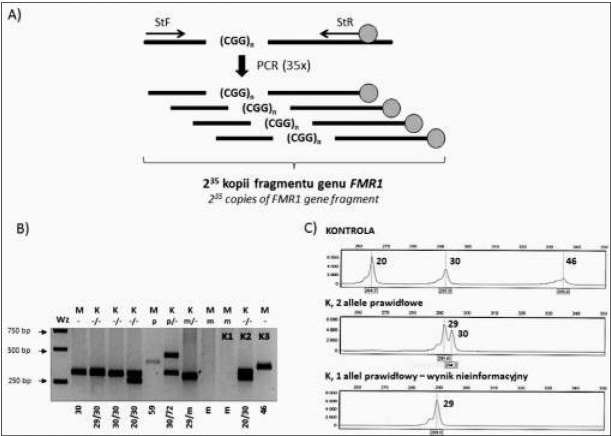
Analiza molekularna genu *FMR1* pod kątem obecności mutacji dynamicznej z wykorzystaniem testu przesiewowego. Test przesiewowy wykonywany jest w oparciu o technikę PCR z wykorzystaniem starterów fluorescencyjnych (a). Produkty amplifikacji rozdzielane są w żelu agarozowym z dodatkiem bromku etydyny (b) lub w sekwenatorze kapilarnym (c). Technika umożliwia identyfikację alleli z zakresu prawidłowego oraz premutacji (do 130 powtórzeń CGG). Razem z próbkami badanymi (1-8) amplifikowane są kontrole (K1 – pełna mutacja, K2 – kontrola prawidłowa, żeńska, 20 i 30 powtórzeń CGG, K3 – kontrola, allel z zakresu „szarej strefy” męska (46 powtórzeń CGG). Oznaczenia: Wz – marker wielkości 1kb (ThermoFisher), „-” – allel z zakresu prawidłowego, p – allel z zakresu premutacji, m – allel z zakresu pełnej mutacji, M – płeć męska, K – płeć żeńska. Na rycinie podano liczbę powtórzeń CGG. Fig. 1. Molecular analysis of dynamic mutation in the FMR1 gene with pre-screening PCR test. The pre-screening test is performed with PCR technique and fluorescently labeled primers (a). The amplification products are resolved in agarose gels with ethidium bromide (b) or by capillary electrophoresis (c). The pre-screening test allows to identify normal or low (up to 130 CGG repeats) premutation alleles. Together with patient samples (1-8), the appropriate controls are amplified (K1 – full mutation, K2 – normal female control with 20 and 30 CGG repeats, K3 – “grey zone” (46 CGG repeats) male control). Legend: Wz – size marker 1kb (ThermoFisher), „-” – normal allele, p – premutation allele, m – full mutation allele, M – male, K – female. The number of CGG repeats is shown.

Uzyskany produkt reakcji PCR rozdzielany jest w żelach agarozowych z dodatkiem bromku etydyny (Rycina 1b) lub w sekwenatorach kapilarnych, jeśli do ampli&kacji wykorzystano startery znakowane fluorescencyjnie (Rycina 1c).

Podstawową wadą rozdziału produktów w żelach agarozowych jest brak możliwości jednoznacznego określenia wielkości alleli. Staje się to problematyczne, zwłaszcza w przypadku uzyskania produktów o większej długości niż stosowane kontrole o znanej liczbie powtórzeń CGG (np. allele z zakresu szarej strefy), pojawienia się rozmytych prążków na żelu, co może świadczyć o obecności mutacji bądź uzyskanie tylko jednego prążka podczas analizy materiału pochodzącego od kobiet. Stąd też jako uzupełnienie takiej analizy stosuje się rozdział fragmentów DNA w sekwenatorze kapilarnym (metoda GeneScan), co umożliwia dokładną ocenę liczby powtórzeń CGG z dokładnością, co do jednego powtórzenia w zakresie alleli prawidłowych oraz niskich premutacji. Wielkość fragmentów szacowana jest na podstawie markera wielkości (np. GeneScan 500 ROX Dye Size Standard, / ermo&sher Scientific), którego **z**adaniem jest umożliwienie automatycznej analizy danych oraz precyzyjnego porównania wielkości fragmentów DNA powstałych w trakcie reakcji PCR [5].

Test przesiewowy umożliwia identy&kację prawidłowych alleli genu *FMR1* i niskich premutacji (<130). Dla mężczyzn posiadających prawidłowy allel i kobiet heterozygot pod względem alleli prawidłowych po przeprowadzeniu analizy wydawany jest wynik informacyjny, prawidłowy. Wyklucza on rozpoznanie zespołu łamliwego chromosomu X w większości przypadków ze względu na niskie prawdopodobieństwo wystąpienia mozaikowatości komórkowej (somatycznej) lub innych mutacji w genie *FMR1*, których identy&kacja nie jest możliwa tą techniką ze względu na ograniczenia procedury diagnostycznej. Jeśli istnieje silne podejrzenie zespołu FXS u pacjenta, badanie należy rozszerzyć pod kątem identy&kacji potencjalnej mozaikowości (badanie z wykorzystaniem technik umożliwiających identy&kację mutacji, badanie różnych tkanek) lub obecności innych zmian w genie *FMR1* [2, 5].

Test przesiewowy nie umożliwia identy&kacji alleli z zakresu mutacji i wysokich (>130) premutacji, jak również nie pozwala na jednoznaczną interpretację wyniku w przypadku kobiet homozygot pod względem alleli z zakresu prawidłowego. Stwierdzenie braku produktu ampli&kacji genu *FMR1* u mężczyzn lub obecności tylko jednego, prawidłowego allela u kobiet stanowi podstawę do wydania wyniku nieinformacyjnego i jest wskazaniem do rozszerzania diagnostyki z wykorzystaniem innych, bardziej czułych metod.

### 2.2 Hybrydyzacja genomowa metodą Southerna

Opisując techniki stosowane w diagnostyce zespołu łamliwego chromosomu X należy wspomnieć o coraz rzadziej stosowanej technice hybrydyzacji genomowej metodą Southerna. Technika została opracowana w 1975 r. i przez długi czas stanowiła podstawę analiz molekularnych w chorobach *FMR1* – zależnych2W Zakładzie Genetyki Medycznej IMiD, który jest laboratorium referencyjnym w zakresie diagnostyki zespołu łamliwego chromosomu X, metoda hybrydyzacji genomowej została w 2014 zastąpiona testem wykonywanym metodą Triplet-Primed PCR. Tabela I. Podsumowanie metod molekularnych stosowanych w diagnostyce zespołu łamliwego chromosomu X. [2].

Hybrydyzacja genomowa typu Southern umożliwiała nie tylko identy&kację wysokich premutacji, pełnych mutacji i mozaikowatości, lecz również, dzięki zastosowaniu metylowrażliwego enzymu restrykcyjnego, pozwalała na określenie statusu metylacji w *locus FMR1*. Informacja o statusie metylacji była szczególnie przydatna w sytuacji, gdy trzeba było rozróżnić wysoką premutację i pełną mutację z zakresu ok. 200 powtórzeń CGG [2].

Mimo tego, że metoda wydaje się być odpowiednia do kompleksowej diagnostyki zespołu łamliwego chromosomu X, to ma ona swoje ograniczenia, przez co została wyparta przez nowocześniejsze metody molekularne. Główną wadą metody jest duża ilość (10 μg) wysokocząsteczkowego DNA o wysokiej jakości, niezbędnego do przeprowadzenia badania. W badaniach własnych w około 5% przypadków odstępowano od wykonania diagnostyki ze względu na brak odpowiedniej ilości DNA, do czasu ponownego pobrania materiału od osoby badanej. Ponadto metoda jest pracochłonna i długotrwała – cała procedura od trawienia DNA enzymami restrykcyjnymi do momentu wizualizacji wyników trwa około 5 dni roboczych (Rycina 2a) [17].

**Ryc. 2:**
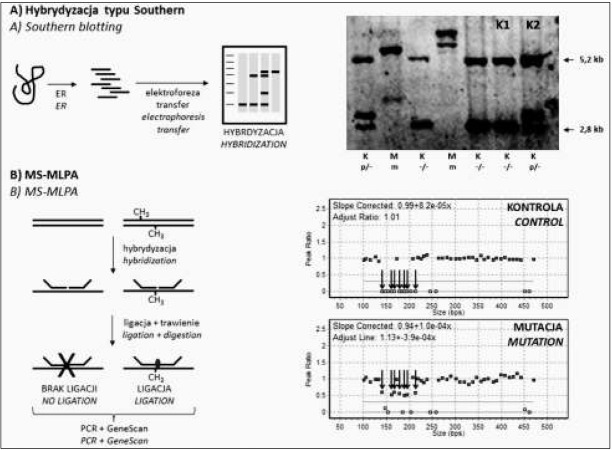
Metody wykorzystywane w diagnostyce zespołu łamliwego chromosomu X uwzględniające analizę metylacji w badanym regionie: hybrydyzacja typu Southern (A) i MS-MLPA (B). A) DNA trawione jest enzymami restrykcyjnymi (*EcoRI* i wrażliwy na metylację *NruI*) i rozdzielane w żelu agarozowym. Rozdzielony DNA przenoszony jest na membranę nitrocelulozową (transfer), a następnie prowadzona jest hybrydyzacja z sondą specyficzną dla *locus FMR1*. Kolejnym etapem jest detekcja sondy związanej z DNA i analiza wzoru prążków. Na obrazie widać dwa zakresy fragmentów DNA: pierwszy o wielkości ≥2,8kpz odpowiadający sekwencjom niemetylowanym (allele prawidłowe i z zakresu premutacji) oraz drugi ≥5,2kpz odpowiadający sekwencjom metylowanym (allele z zakresu mutacji, u kobiet dodatkowo fragmenty odpowiadające allelom znajdującym się na inaktywowanym chromosomie X). Oznaczenia: „-” – allel z zakresu prawidłowego, p – allel z zakresu premutacji, m – allel z zakresu pełnej mutacji, M – płeć męska, K – płeć żeńska. B) Metoda MS-MLPA jako modyfikacja klasycznej metody MLPA, umożliwia jednoczesną ocenę liczby kopii badanego regionu i analizę metylacji; technika wykorzystuje działanie metylowrażliwego enzymu restrykcyjnego *HhaI* – sekwencje niemetylowane są trawione enzymem restrykcyjnym, co uniemożliwia ligację sond specyficznych względem badanego regionu i skutkuje brakiem produktu amplifikacji. Badanie, wiarygodne jedynie w przypadku pacjentów płci męskiej, prowadzone jest z wykorzystaniem zestawu ME029 zawierającego m.in. sondy specyficzne dla genu *FMR1*. U osób płci męskiej z prawidłowym allelem (kontrola), gen *FMR1* jest niemetylowany co jest równoznaczne z brakiem sygnału dla wybranych sond (strzałki). U pacjentów z pełną mutacją, gen jest metylowany, w związku z czym obserwuje się sygnał dla sond specyficznych względem genu *FMR1* (w tym przypadku mamy do czynienia z mozaiką metylacyjną – metylowanych jest ok. 50% alleli) Fig. 2. Techniques used in the molecular diagnosis based on the methylation analysis of the examined region: Southern blotting (A) and MS-MLPA (B). A) DNA is digested with restriction enzymes (EcoRI and methylation-sensitive NruI), resolved in agarose gel and then transferred to the nitrocellulose membrane. Next the hybridization is performed with probe specific for FMR1 locus. Finally, the probe detection, visualization and analysis of band pattern are performed. The hybridization signals two ranges of DNA fragments are present: first ≥2,8kbp that corresponds to unmethylated sequences (normal and premutation alleles) and second ≥5.2kbp that corresponds to methylated sequences (full mutation alleles, in females DNA fragments that match to alleles on inactivated X chromosome). Legend: „-” – normal allele, p – premutation allele, m – full mutation allele, M – male, K – female. B) MS-MLPA analysis as a modification of classic MLPA technique allows to identify copy number variation and methylation defects in single reaction; this technique uses HhaI – a methyl-sensitive restriction enzyme that digests unmethylated DNA. Therefore, the probes specific for the examined region cannot bind to their target sequences and be amplified. The analysis with MS-MLPA and ME029 kit, that contains probes specific for FMR1 gene, is only reliable in male patients. In male patients with normal allele (control), the FMR1 gene is unmethylated therefore there is no amplification for probes specific to methylated FMR1 sequences (indicated by arrows). In patients with full mutation, the FMR1 gene is usually methylated, so amplification of specific probes is present. In presented case, the methylation mosaicism is present (about 50% of methylated alleles).

### 2.3 Triplet-Primed PCR (TP-PCR)

Jedną z metod, które wyparły hybrydyzację genomową typu Southern jest metoda TP-PCR (ang. *tripled-primed PCR*) opracowana do analiz wykonywanych pod kątem obecności mutacji dynamicznych. Znalazła ona zastosowanie nie tylko w diagnostyce zespołu łamliwego chromosomu X, lecz również innych chorób związanych z obecnością mutacji dynamicznej, takich jak: dystro&a miotoniczna, choroba Huntingtona czy choroba Friedricha. Metoda ta została opracowana przez Warnera i współpracowników w 1996 roku w celu analizy ekspansji sekwencji CAG w genie *DMPK*, którego mutacja jest odpowiedzialna za wystąpienie dystro&i miotonicznej typu 1. Pierwszy test tego typu dedykowany dla genu *FMR1* został opisany w 2006 roku przez firmę Abbott [18].

Metoda TP-PCR umożliwia rozwiązanie wielu problemów technicznych ograniczających dotychczas możliwość dokładnej oceny liczby powtórzeń CGG przy zastosowaniu standardowej reakcji ampli&kacji i analizy GeneScan. Podstawą metody jest zastosowanie dodatkowego, poza starterami obejmującymi sekwencje CGG, specyficznego startera, który jest komplementarny do regionu CGG i może przyłączyć się w dowolnym miejscu ciągu powtórzeń trójki nukleotydowej (Rycina 3). Startery stosowane w reakcji są wyznakowane fluorescencyjnie, dzięki czemu możliwy jest rozdział produktów reakcji w sekwenatorze kapilarnym [19]. W wyniku reakcji otrzymuje się specy&czny produkt odpowiadający ampli&kacji fragmentu zawierającego powtórzenia CGG z zastosowaniem standardowych starterów oraz amplikony o różnej długości pochodzące z ampli&kacji z wykorzystaniem startera specy&cznego względem sekwencji CGG3Metoda wykorzystywana jest w zestawach: FMR1 TP-PCR and Sizing PCR (Abbott),FragilEase™ PCR assay (Perkin-Elmer), FastFraX FMR1 Identi&cation Kit and FastFraX FMR1 Sizing Kit (TNR Diagnostics) i AmplideX® PCR/CE FMR1 Kit (Asuragen).. W reakcji wykorzystuje się specjalną mieszankę polimeraz, która umożliwia identy&kację pełnego zakresu alleli genu, z dokładną oceną liczby powtórzeń CGG w zakresie do 200, a także amplifikację mutacji. Metoda umożliwia także analizę powtórzeń w genie *FMR1* pod kątem obecności sekwencji przerywającej AGG, co może mieć znaczenie w ocenie ryzyka ekspansji niestabilnych powtórzeń [20, 21].

**Ryc. 3:**
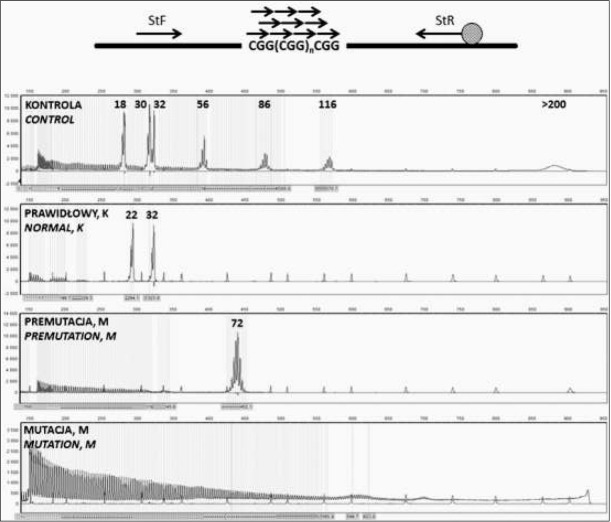
Analiza molekularna genu *FMR1* z wykorzystaniem metody TP-PCR: schemat reakcji i przykładowe wyniki analizy. Jako kontrola wykorzystywana jest mieszania fragmentów odpowiadających allelom z zakresu prawidłowego (18, 30 i 32 powtórzenia CGG), premutacji (56, 86 i 116 powtórzeń CGG) i pełnej mutacji. Oznaczenia: K – płeć żeńska, M – płeć męska Fig. 3. Molecular analysis of FMR1 gene with TP-PCR method: the reaction scheme and examples of analysis results. As a control, a mixture of fragments corresponding to normal (18, 30 and 32 CGG repeats), premutation (56, 86 and 116 CGG repeats) and full mutation alleles. Legend: K – female, M – male

Z danych literaturowych wynika, że metoda TP-PCR jest techniką o wysokiej czułości i specyficzności, która w sposób powtarzalny umożliwia zidenty&kowanie mutacji o dużej liczbie powtórzeń CGG i wykazuje wysoką zgodność z metodą hybrydyzacji genomowej typu Southerna. Wysoka czułość metody pozwala także na identy&kację mozaikowości somatycznej (od ok. 10%) oraz potwierdzenie homozygotyczności u kobiet posiadających allele z zakresu prawidłowego [22].

### 2.4 Metylo-specyficzna multipleksowa amplifikacja sond zależna od ligacji (MS-MLPA)

Technika MS-MLPA (ang. *Methylation-Specitic Multiplex-Ligation-dependent Probe Amplification*) jest metodą półilościową i stanowi mody&kację klasycznej metody MLPA. Metoda, poza oceną liczby kopii w badanym regionie chromosomowym (analiza copy numer variation, CNV), umożliwia także analizę statusu metylacji w określonych *loci*, dzięki zastosowaniu metylowrażliwego enzymu restrykcyjnego *HhaI* (Rycina 2b) [23].

Metoda MS-MLPA została opracowana przez firmę MRC-Holland, która jest również wyłącznym dystrybutorem swoich odczynników. Zestaw do analizy metylacji *FMR1* (zestaw ME029) umożliwia tylko analizę metylacji i CNV, nie pozwala natomiast na określenie wielkości allelu/alleli i liczby powtórzeń CGG w badanym regionie. Ograniczeniem metody jest również fakt, że jest ona dedykowana wyłącznie do analizy materiału od pacjentów płci męskiej. Wynika to z faktu, że u kobiet dochodzi do nielosowej inaktywacji chromosomu X związanej z jego metylacją. Powoduje to, że analiza metylacji genów zlokalizowanych na chromosomie X daje w przypadku próbek od osób płci żeńskiej niejednoznaczne i trudne do właściwej interpretacji wyniki.

Zaletą metody, podobnie jak w przypadku TP-PCR, jest niewielka ilość DNA potrzebna do wykonania badania molekularnego (50-250 ng). W odróżnieniu od alternatywnych technik stosowanych w analizie metylacji (np. MS-PCR), reakcja MS-MLPA nie wymaga mody&kacji DNA kwaśnym dwusiarczynem sodowym, co znacznie skraca czas badania. Analizy wykonywane z wykorzystaniem metody MLPA i jej mody&kacje są stosunkowo łatwe do przeprowadzenia, jak również umożliwiają szybkie otrzymanie wyników (do 48 h). W literaturze naukowej jest wiele publikacji, które potwierdzają skuteczność i użyteczność techniki MS-MLPA w określaniu statusu metylacji różnych genów związanych zarówno z chorobami typowo genetycznymi np. FXS, zespół Angelmana czy Pradera-Williego jak i licznymi chorobami nowotworowymi [24].

### 2.5 Alternatywne techniki stosowane w analizie chorób *FMR1*-zależnych

Na potrzeby analizy metylacji genu *FMR1* opracowano modyfikacje techniki TP-PCR lub PCR. Pierwsza z nich4Wykorzystywana w zestawie FastFraX™ FMR1 Methylation Status Kit (TNR Diagnostics). wykorzystuje modyfikację DNA genomowego wodorosiarczynem sodu, w trakcie, której niemetylowane cytozyny przekształcane sąw uracyl. Dzięki temu zastosowanie starterów specyficznych względem sekwencji metylowanych i niemetylowanych umożliwia identyfikację pełnego zakresu alleli w genie *FMR1* [25]. W drugim przypadku5Wykorzystywana w zestawie AmplideX® mPCR FMR1 (Asuragen). wykorzystywany jest metylowrażliwy enzym restrykcyjny *HpaII*, którym trawiony jest DNA pacjenta. Następnie jednocześnie na próbce trawionej enzymem *HpaII* i nietrawionej prowadzona jest reakcja PCR z zastosowaniem starterów fluorescencyjnych. W obydwu przypadkach, wyniki dwóch reakcji PCR porównywane są do siebie, na podstawie czego można ocenić który allel i w jakim stopniu ulega metylacji. W przeciwieństwie do metody MS-MLPA, obydwie te techniki umożliwiają również analizę próbek pochodzących od osób płci żeńskiej [26].

Podjęto także próby wykorzystania krzywych topnienia produktów reakcji TP-PCR do oceny metylacji i zakresu alleli w genie *FMR1*. Reakcja amplifikacji przeprowadzana jest w termocyklerze z opcją monitorowania fluorescencji w czasie rzeczywistym (np. LightCycler Real-Time PCR System, Roche Applied Science) z wykorzystaniem barwnika SYBRGreen. Produkt reakcji PCR jest stopniowo denaturowany poprzez powolny wzrost temperatury od 60 do 95°C z jednoczesnym monitorowaniem spadku fluorescencji wskutek uwalniania barwnika SYBRGreen z dwuniciowych fragmentów DNA. Krzywe topnienia różnią się między sobą, w zależności od liczby powtórzeń CGG, co umożliwia odróżnienie alleli prawidłowych od tych z premutacją i pełną mutacją. Założeniem twórców metody była możliwość jej wykorzystania jako testu przesiewowego, dlatego nie nadaje się ona do dokładnej oceny liczby powtórzeń CGG w genie *FMR1*, a jedynie pozwala na predykcję obecności alleli nieprawidłowych [27].

Ograniczeniem wszystkich powyżej zaprezentowanych metod stosowanych w diagnostyce chorób *FMR1*-zależnych jest brak możliwości oceny dokładnej liczby powtórzeń w allelach z zakresu pełnej mutacji. W dużym przybliżeniu pozwalała na to metoda hybrydyzacji typu Southern, jednak jest ona stopniowo wypierana przez szybsze i mniej wymagające metody molekularne. Dokładną ocenę liczby powtórzeń w przypadku pełnej mutacji umożliwiło dopiero wprowadzenie technologii sekwencjonowania następnej generacji, zwłaszcza technologii SMRT (ang. single-molecule real-time), która nie wymaga wcześniejszej amplifikacji materiału do sekwencjonowania. Sekwencjonowanie pojedynczej cząsteczki DNA jest możliwe dzięki unieruchomieniu pojedynczych cząsteczek polimerazy na specjalnie przygotowanych płytkach reakcyjnych (ang. zero-mode waveguide ZMW) pozwalających na dokładny odczyt nowo zsyntetyzowanej nici kwasu nukleinowego. Długość sekwencji, których odczyt jest możliwy dzięki tej technice, wynosi do 60 tysięcy par zasad (średnia długość 10kb), podczas gdy w przypadku technologii Illumina najdłuższe odczyty mają do 600pz. W przypadku genu *FMR1* możliwy był odczyt sekwencji zawierających ponad 750 powtórzeń CGG (>2,25 kb) oraz dokładne określenie położenia sekwencji AGG. Sugeruje się, że w przyszłości technika ta umożliwi również analizę metylacji w obrębie sekwencji powtórzonych [28].

## 3 Schemat Postępowania Diagnostycznego w Zakładzie Genetyki Medycznej Imid

Jak wspominano wcześniej, badania molekularne w kierunku zespołu łamliwego chromosomu X są jednymi z częściej zlecanych badań diagnostycznych nie tylko w przypadku stwierdzenia NI, lecz również u pacjentów z opóźnieniem rozwoju psychoruchowego, opóźnionym rozwojem mowy czy zaburzeniami zachowania, w tym zaburzeniami ze spektrum autyzmu. Zdarzają się pacjenci, u których zespół łamliwego chromosomu X zostaje potwierdzony, jeszcze przed rozwinięciem pełnego obrazu klinicznego choroby. Istotne jest wdrożenie do codziennej praktyki laboratoriów zajmujących się diagnostyką molekularną dokładnych i efektywnych metod umożliwiających szybką i wiarygodną analizę w kierunku FXS oraz chorób *FMR1*-zależnych.

W Zakładzie Genetyki Medycznej, w przypadku pacjentów z objawami sugerującymi możliwość wystąpienia zespołu łamliwego chromosomu X, pierwszym wykonywanym badaniem jest test przesiewowy PCR z analizą GeneScan (Rycina 4). Prawidłowy wynik tego testu (allel z zakresu prawidłowego u osób płci męskiej, dwa prawidłowe allele u osób płci żeńskiej) jest podstawą do wykluczenia zespołu łamliwego chromosomu X w większości przypadków. W sytuacji, gdy pacjent z ewidentnymi cechami zespołu FXS ma prawidłowy wynik badania przesiewowego, należy rozważyć możliwość wykonania dalszych analiz genu *FMR1* pod kątem obecności delecji obejmujących fragment genu *FMR1* (np. technika MLPA, porównawcza hybrydyzacja genomowa do mikromacierzy, aCGH) lub mutacji punktowych (technika sekwencjonowania metodą Sangera). Jeśli test był wykonany w celu wykluczenia zespołu łamliwego chromosomu X, dalsze procedury diagnostyczne będą zależeć od objawów klinicznych obserwowanych u Badanego i w zależności od tego może zostać podjęta decyzja o analizie celowanej na konkretne rozpoznanie kliniczne lub analizie wysokoprzepustowej (technika aCGH, sekwencjonowanie następnej generacji).

**Ryc. 4:**
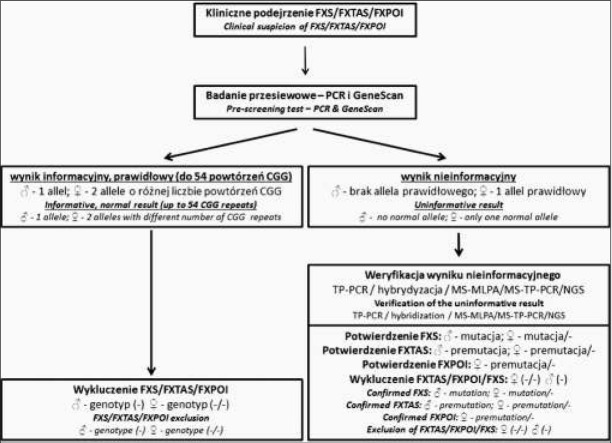
Schemat postępowania diagnostycznego w przypadku podejrzenia FXS, FXTAS lub FXPOI. Fig. 4. The diagnostic workflow in case of clinical suspicion of FXS, FXTAS or FXPOI.

W przypadku identyfikacji u objawowego pacjenta allela z zakresu „szarej strefy”, wskazane jest wykonanie badania molekularnego u jego najbliższych krewnych, w celu oceny czy transmisja tego allela w rodzinie badanego ma charakter stabilny. W przypadkach, które zostały zidentyfikowane w Zakładzie Genetyki Medycznej allele z zakresu szarej strefy miały przynajmniej 2 powtórzenia AGG i były stabilnie przekazywane przez przynajmniej 3 pokolenia.

Nieinformacyjny wynik testu przesiewowego (brak prawidłowego allela u osób płci męskiej, tylko jeden prawidłowy allel u osób płci żeńskiej) jest **zawsze** wskazaniem do rozszerzenia badań molekularnych. W naszym przypadku jako technika umożliwiająca identyfikację alleli z zakresu premutacji/pełnej mutacji stosowana jest metoda TP-PCR wdrożona po okresie jej walidacji względem techniki hybrydyzacji genomowej typu Southern. Identyfikacja mutacji w genie *FMR1* będzie stanowić potwierdzenie rozpoznania klinicznego zespołu łamliwego chromosomu X, zaś premutacji potwierdzenie nosicielstwa zespołu FXS. Badanie z wykorzystaniem techniki TP-PCR jest również zalecane w przypadku badania osób z rodziny pacjenta oraz w przypadku diagnostyki prenatalnej, gdyż umożliwia wykluczenie obecności ewentualnej mozaikowości somatycznej.

W przypadku konieczności analizy metylacji DNA w *locus FMR1* stosowane są techniki MS-MLPA (osoby płci męskiej) lub MS-TP-PCR (osoby płci żeńskiej). Należy jednak pamiętać o tym, że status metylacji DNA w krwi obwodowej (zazwyczaj na tym materiale wykonywane jest badanie) nie musi odpowiadać statusowi metylacji genu *FMR1* w układzie nerwowym, więc ewentualna interpretacja wyniku badania w kontekście obserwowanych objawów klinicznych będzie trudna.

Specyficzną sytuacją jest identyfikacja u pacjentów objawowych allela z zakresu premutacji –w literaturze nie ma doniesień o związku premutacji w genie *FMR1* z rozwojem niepełnosprawności intelektualnej. Zgodnie z wytycznymi EMQN, w takiej sytuacji nie można wykluczyć obecności mozaikowości somatycznej – obecności alleli z zakresu pełnej mutacji w tkankach innych niż badane. Warto wtedy wykonać badanie molekularne stosując DNA wyizolowany z tkanki wywodzącej się z innego listka zarodkowego niż krew (np. fibroblastach skóry). Nasze doświadczenia wskazują jednak, że w przypadku niskich premutacji (<70 powtórzeń CGG), wynik badania materiału z fibroblastów jest również prawidłowy, zaś u jednego pacjenta, po wykonaniu analizy eksomu, stwierdzono obecność mutacji patogennej w innym genie związanym z patogenezą niepełnosprawności intelektualnej (dane własne IMiD). W przypadku wysokich premutacji (>180 powtórzeń CGG) warto jest rozważyć wykonanie analizy metylacji, gdyż obecność objawów klinicznych może również wynikać z inaktywacji genu *FMR1* poprzez metylację.

W przypadku pacjentów z podejrzeniem FXTAS i FXPOI stosowany jest podobny schemat postępowania diagnostycznego (test przesiewowy, test TP-PCR w przypadku wyniku nieinformacyjnego). Aby skrócić czas oczekiwania na wynik informacyjny, analiza może zostać wykonana od razu techniką TP-PCR - zależy to od decyzji lekarza kierującego/pacjenta. Ważne jest natomiast, aby w przypadku decyzji o zleceniu badania przesiewowego, poinformować pacjenta o możliwości uzyskania wyniku nieinformacyjnego i konieczności wykonania dodatkowych badań molekularnych w takim przypadku.

Przedstawiony powyżej schemat postepowania diagnostycznego w przypadku podejrzenia zespołu łamliwego chromosomu X i innych chorób *FMR1*-zależnych ma jedynie charakter informacyjny. Jest on jednak zgodny z zaleceniami European Molecular Genetics Quality Network (EMQN). Odzwierciedleniem tego są pozytywne wyniki Zakładu Genetyki Medycznej w testach kontroli jakości organizowanych przez EMQN, jak również fakt że przez wiele lat Zakład pozostawał ośrodkiem referencyjnym w zakresie diagnostyki zespołu łamliwego chromosomu X.

### Podziękowania

*Pragniemy serdecznie podziękować Pracownikom Zakładu Genetyki Medycznej IMiD, którzy uczestniczyli w diagnostyce i badaniach naukowych nad zespołem łamliwego chromosomu X oraz innych zaburzeń intelektualnych o podłożu genetycznym, którzy nie są współautorami tej pracy. Prace naukowe dotyczące niepełnosprawności intelektualnej sprzężonej z chromosomem X były możliwe m.in. dzięki finansowaniu z Narodowego Centrum Nauki (nr grantu: 2012/07/B/NZ4/01764)*.

## References

[1] Sielska D, Milewski M, Bal J (2002). Molekularna patogeneza zespołu łamliwego chromosomu X. Med Wieku Rozwoj.

[2] Sielska D, Milewski M (2006). Analiza molekularna w zespole łamliwego chromosomu x (Frax).

[3] Gos M (2017). Epigenetyka. rozdział w Genetyka medyczna i molekularna, pod red. J. Bal. PWN Warszawa.

[4] Landowska A, Rzońca S, Bal J, Gos M (2018). Zespół łamliwego chromosomu X i choroby FMR1-zależne – objawy kliniczne, epidemiologia i podłoże molekularne choroby. Dev Period Med.

[5] Rzońca SO, Gos M, Szopa D (2016). Towards a Better Molecular Diagnosis of FMR1-Related Disorders − A Multiyear Experience from a Reference Lab. Genes (Basel).

[6] Milewski M, Bal J (1993). Mutacje dynamiczne. Rola niestabilnych sekwencji DNA w niektórych chorobach genetycznych człowieka. Postępy Biochemii.

[7] Milewski M (1994). Molekularne podłoże zespołu łamliwego chromosomu X. Ped Pol.

[8] Milewski M, Bal J, Mazurczak T (1996). Między pokoleniowe przekazywanie mutacji warunkujących Zespół łamliwego chromosomu X. Ped Pol.

[9] Yrigollen CM, Durbin-Johnson B, Gane L (2012). AGG interruptions within the maternal FMR1 gene reduce the risk of offspring with fragile X syndrome. Genet Med.

[10] Zoll B, Bal J, Pohnke J, Bockel B, Sancken U, Knobloch O (1992). Martin-Bell-Syndrom. Verbesserte Moglichkeiten bei der molekulargenetischen Diagnostik. Monatsschr Kinder heilkd.

[11] Milewski M, Żygulska M, Bal J, Deelen WH, Obersztyn E, Bocian E, Halley DJJ, Horst J, Mazurczak T (1996). Analysis of unstable DNA sequence in FMR-1 gene in families of Polish origin with fragile X syndrome. Acta Biochem Pol.

[12] Milewski M, Bal J, Bocian E, Obersztyn E, Mazurczak T (1996). Relationship between molecular, cytogenic and clinical parameters in 61 individuals with full mutation in FMR1 gene. J Appl Genet.

[13] Mazurczak T, Bocian E, Milewski M, Obersztyn E, Stańczak H, Bal J, Szamotulska K, Karwacki M (1996). Frequency of Fra X Syndrome among institionalized mentally retarded males in Poland. Amer J Med Gent.

[14] Mazurczak T, Karwacki MW, Obersztyn E, Palczewska I, Milewski M, Bocian E (2000). Diagnostyka kliniczna zespołu łamliwego chromosomu X– wyniki badań klinicznych, cytogenetycznych i molekularnych 109 nosicieli mutacji w genie FMR1. Neurologia Dziecięca.

[15] Hagerman RJ, Leehey M, Heinrichs W (2001). Intention tremor, parkinsonism, and generalized brain atrophy in male carriers of fragile X. Neurology.

[16] Biancalana V, Glaeser D, McQuaid S (2014). EMQN best practice guidelines for the molecular genetic testing and reporting of fragile X syndrome and other fragile X-associated disorders. Eur J Hum Genet.

[17] Tzeng C-C, Liou C-P, Chien-Feng Li C-F (2009). Methyl-CpG-Binding PCR of Bloodspots for Confirmation of Fragile X Syndrome in Males.

[18] Moeschler JB, Shevell M (2014). Comprehensive Evaluation of the Child With Intellectual Disability or Global Developmental Delays. Pediatrics.

[19] Das Bhowmi A, Rangaswamaia S, Srinivas G (2015). Molecular genetic analysis of trinucleotide repeat disorders (TRDs) in Indian population and application of repeat primed PCR. Eur J Med Genet.

[20] Hantash FM, Goos DG, Tsao D (2010). Qualitative assessment of FMR1 (CGG)n triplet repeat status in normal, intermediate, premutation, full mutation, and mosaic carriers in both sexes: Implications for fragile X syndrome carrier and newborn screening. Genet Med.

[21] Chen L, Hadd A, Sah S (2010). An Information-Rich CGG Repeat Primed PCR That Detects the Full Range of Fragile X Expanded Alleles and Minimizes the Need for Southern Blot Analysis. J Mol Diagn.

[22] Rajan-Babu I-S, Law H-Y, Yoon C-S (2015). Simplified strategy for rapid first-line screening of fragile X syndrome: closed-tube triplet-primed PCR and amplicon melt peak analysis. Expert Rev Mol Med.

[23] Gatta V, Gennaro E, Franchi S (2013). MS-MLPA analysis for FMR1 gene: evaluation in a routine diagnostic setting. BMC Medical Genetics.

[24] Majchrzak A (2009). Baer-Dubowska W Markery epigenetyczne w diagnostyce: Metody oceny metylacji DNA. Diagn Lab.

[25] Zhou Y, Lum JM, Yeo G-H (2006). Simplified Molecular Diagnosis of Fragile X Syndrome by Fluorescent Methylation-Specific PCR and GeneScan Analysis. Clin Chem.

[26] Grasso M, Boon EM, Filipovic-Sadic S (2014). A novel methylation PCR that offers standardized determination of FMR1 methylation and CGG repeat length without southern blot analysis. J Mol Diagn.

[27] Teo CR, Law HY, Lee CG (2012). Screening for CGG repeat expansion in the FMR1 gene by melting curve analysis of combined 5’ and 3’ direct triplet-primed PCRs. Clin Chem.

[28] Loomis EW, Eid JS, Peluso P (2013). Sequencing the unsequenceable: Expanded CGG-repeat alleles of the fragile X gene. Genome Res.

